# Association between gross motor function (GMFCS) and manual ability (MACS) in children with cerebral palsy. A population-based study of 359 children

**DOI:** 10.1186/1471-2474-8-50

**Published:** 2007-06-21

**Authors:** Katharina Delhusen Carnahan, Marianne Arner, Gunnar Hägglund

**Affiliations:** 1 Department of Orthopaedics, Helsingborg Hospital, S 251 87 Helsingborg, Sweden; 2 Department of Orthopaedics, Lund University Hospital, S 221 85 Lund, Sweden

## Abstract

**Background:**

The Gross Motor Function Classification System (GMFCS) has become an important tool to describe motor function in children with Cerebral Palsy (CP). The Manual Ability Classification System (MACS) was developed recently as a corresponding classification of manual ability. The aim of this study was to describe the association between gross motor function and manual ability in a total population of children with cerebral palsy.

**Methods:**

365 children, born 1992 to 2001, who were registered in a population-based health care programme (CPUP) for children with CP living in the south of Sweden were included in the study. GMFCS was evaluated by the child's physiotherapist and MACS by the occupational therapist. CP diagnosis and subtype were determined by the neuropaediatrician at or after the age of four.

**Results:**

GMFCS levels were available in all 365 children, MACS levels in 359 (98%). There was a poor overall correlation between gross motor function and manual ability. However, different associations between gross motor function and manual ability were found in the different diagnostic subtypes. Children with spastic hemiplegia generally had a lower level of manual ability than gross motor function (p < 0.001). The reverse association was generally found in children with spastic diplegia (p < 0.001). Children with dyskinetic CP had large limitations in both gross motor function and manual ability, with no significant discrepancy between GMFCS and MACS levels.

**Conclusion:**

Gross motor function and manual ability are often discrepant in children with CP, and the patterns seem to vary across the different subgroups based on the predominant neurological findings. To give a complete clinical picture when evaluating these children, both aspects have to be described. The GMFCS and the MACS seem to work well in this context and seem very useful in population-based studies, in health care registers for children with CP, and in clinical practice.

## Background

Cerebral Palsy (CP) is the commonest cause of physical disability in early childhood. It has been defined as a group of motor impairment syndromes secondary to a defect or lesion of the immature brain [1], and children with CP display a variety of functional limitations of varying severity [2,3]. Associated cognitive, visual and other impairments are common and the proposed new definition of CP puts more focus on the activity restrictions and disability [4].

The Swedish classification (SC) of clinical CP subtypes [5] has been accepted and used internationally, but recently the Surveillance of Cerebral Palsy in Europe (SCPE) group proposed a new classification of CP subtypes [6,7]. In the SCPE classification, spastic CP is divided only into bilateral and unilateral (hemiplegia). The SC subtypes spastic tetraplegia and diplegia are thus not separated, instead children are described according to functional level in lower and upper extremities, cognitive development, visual function, hearing, epilepsy etc.

The GMFCS was developed to describe gross motor function in children with CP and has its focus on self-initiated movements, in particular sitting and walking [8]. It is an age-related five-level system in which level I represents the least limitation and level V the most (Table I). The GMFCS has proved to be a valid and reliable tool [8] and has been reported to remain relatively stable over time [9-11]. The GMFCS has been internationally accepted and is widely used. According to the designers of the GMFCS, most children will remain at the same level from age 2 to 12 years, which makes it possible to try to predict gross motor development [12].

**Table 1:** Summary of the criteria for the Gross Motor Function Classification System (GMFCS) and the Manual Ability Classification System (MACS)

*GMFCS*	*MACS*
Level I Walks without restrictions, limitations in more advanced gross motor skills	Level I Handles objects easily and successfully
Level II Walks without restrictions, limitations walking outdoors and in the community	Level II Handles most objects but with somewhat reduced quality and/or speed of achievement
Level III Walks with assistive mobility devices, limitations walking outdoors and in community	Level III Handles objects with difficulty; needs help to prepare and/or modify activities
Level IV Self mobility with limitations, children are transported or use power mobility outdoors and in the community	Level IV Handles a limited selection of easily managed objects in adapted situations
Level V Self mobility is severely limited, even with use of assistive technology	Level V Does not handle objects and has very limited ability to perform even simple actions

Describing upper limb function in CP has been a more challenging task. A classification of bimanual fine function (BFMF) was described by Beckung et al in 2002 [13]. In the BFMF, manipulation and gripping ability in both hands is classified in a five-level system. Data on validity and reliability of the BFMF has to date not been published. A new classification called the Manual Ability Classification System was very recently developed [14]. The MACS classifies how well children aged 4–18 years with CP use their hands when handling objects in daily activities. It is designed to reflect the child's typical manual performance, not the maximal capacity. The focus is on manual ability as defined in the International Classification of Functioning, Disability and Health [15] and the MACS level is influenced by environmental and personal factors. Like the GMFCS, the MACS is a five-level system where level I represents the best manual ability and level V indicates that the child does not have any active hand function (Table 1). Good validity and reliability have been reported [14,16], and the MACS has already gained much international attention. It has to date been translated into 13 languages [17].

In the health care programme and register for children with CP in Sweden, CPUP, both GMFCS and MACS are used and form the basis for our secondary prevention protocol. The main goal for CPUP is to prevent hip dislocation and severe contractures, and the programme has so far been successful [18-20].

The aim of the present study was to explore the relationship between gross motor function and manual ability as measured by the GMFCS and the MACS, respectively, in a defined total population of children with CP.

## Methods

In 1994, a register and a health care programme for children with cerebral palsy (CPUP) was started in southern Sweden [18,19]. The register includes all children with CP born after 1 January 1990 living in the counties of Skåne and Blekinge, which have a total population of about 1.3 million. Since 2005, CPUP is a national health care register approved by the National Board of Health and Welfare in Sweden. The programme includes a continuing standardised follow-up of passive joint motion, gross and fine motor function, clinical findings and treatment. The local physiotherapist and occupational therapist examine the children twice a year until the age of six and then once a year. The data from the last examination (2006) of each child in the CPUP register were collected in the present study.

The total material comprised all 398 children with CP in the area born 1992–2001. When the MACS was introduced in the programme, only children born 1992 and later were classified. Classification according to the MACS can be done at four years of age at the earliest.

**Figure 1:**
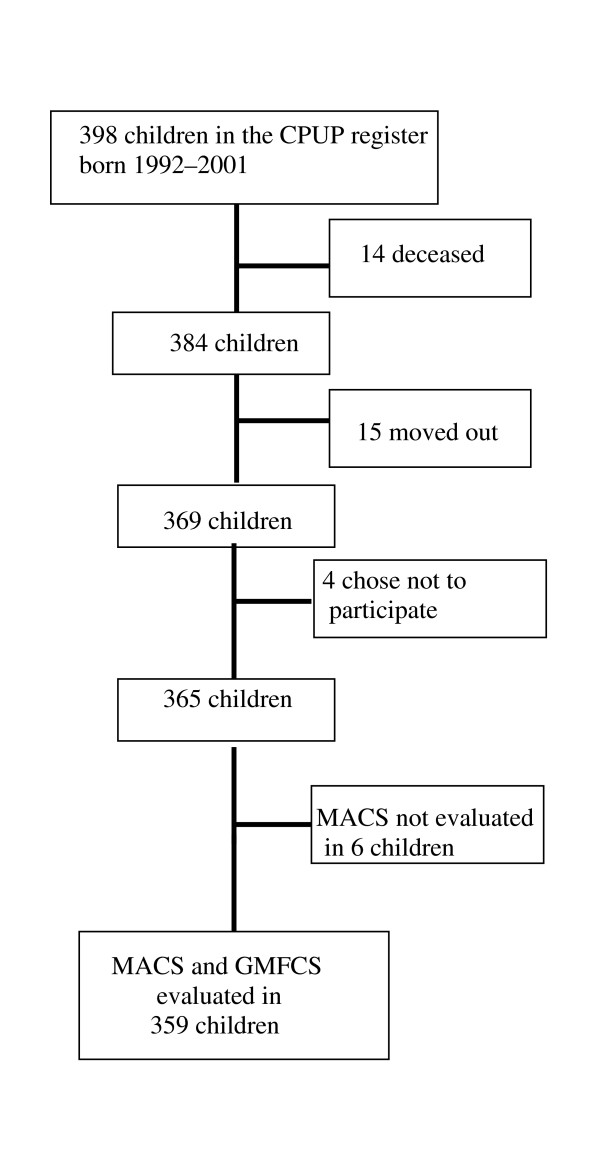
Sample selection and recruitment.

At the time of evaluation, 14 children with severe impairments had died and were for that reason excluded. Four of these children were classified as level V, according to both the MACS and the GMFCS. Eight of the children were classified as GMFCS V and one as GMFCS IV, but these children had died before the introduction of MACS. One child had died before classification of both GMFCS and MACS. A further 15 children had moved out of the area, and 11 of these children moved out before the MACS classification was introduced. The distribution of GMFCS and MACS in these 15 children is presented in Table 2. Four parents chose not to participate in the programme; two of these children were classified as GMFCS I and two as GMFCS II. The remaining 365 children were all classified according to the GMFCS, but 6 children (1,6%) had not been classified according to the MACS. In 359 children, 209 boys (58%), both GMFCS and MACS level was thus documented and these children constitute the study group (Figure 1).

**Table 2:** Distribution of GMFCS and MACS levels in 15 children that had moved out from the study area. NC = Not classified.

GMFCS	MACS	No of children
I	I	1
I	II	1
I	NC	6
III	V	1
III	NC	4
IV	NC	1
V	IV	1

The CP subtypes were classified according to the Swedish Classification (SC) [5] by the child's neuropaediatrician at the age of four years or later. The SC subtypes were transformed to the SCPE system by the authors. The GMFCS level was classified by the child's physiotherapist and the MACS level by the child's occupational therapist according to the available manuals for the GMFCS and the MACS. Both classification systems are available in Swedish. The level of GMFCS and MACS was determined at the same age for each child and the most recent evaluation was used.

### Statistics

We calculated the non weighted Kappa statistics as a measure of the overall agreement between GMFCS and MACS. According to Altman [21] the kappa value is to be interpreted as follows: < 0.20 as poor agreement, 0.21–0.40 as fair, 0.41–0.60 as moderate, 0.61–0.80 as good and > 0.80 as very good agreement. The Wilcoxon signed ranks test and the Sign test were used to analyse systematic differences in the diagnostic subgroups A level of p < 0.05 was regarded as statistically significant. The statistical analysis was done with SPSS 12.01 for Windows (SPSS Inc).

## Results

The distribution between CP subtypes according to the SC and the SCPE systems is shown in Table 4. Of the total sample of 359 children, 283 (79%) had a spastic type of CP, 51 (14%) a dyskinetic and 7 (2%) an ataxic type. Of the 283 children with the spastic type, 121 had a unilateral and 162 a bilateral type according to the SCPE system.

**Table 4:** Correlation between Manual Ability Classification System (MACS) and Gross Motor Function Classification System (GMFCS) level in 359 children with cerebral palsy.

	MACS levels					
		
	**I**	**II**	**III**	**IV**	**V**	**Total**
GMFCS levels						
**I**	112	49	9	2	-	172
**II**	22	14	11	-	1	48
**III**	14	12	11	8	1	46
**IV**	2	5	10	17	17	51
**V**	1	-	2	4	35	42
**Total**	151	80	43	31	54	359

**Figure 2:**
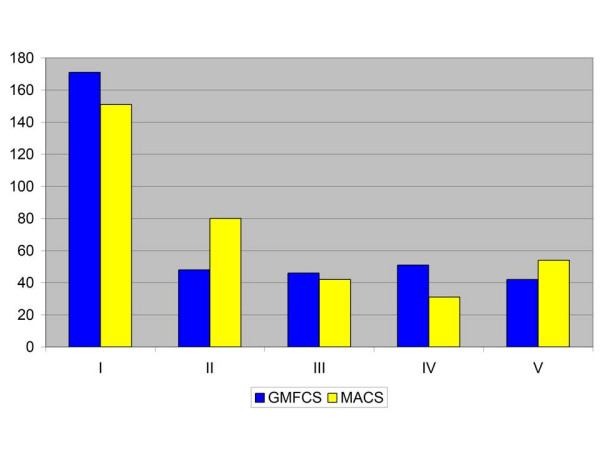
Distribution of GMFCS and MACS levels in the total population of children with CP.

The distribution between levels of gross motor function (GMFCS) and manual ability (MACS) is shown in Figure 2 and Table 4.

The highest level of both gross motor function (GMFCS I) and manual ability (MACS I) was seen in 112 children (31%). There were 220 children (61%) who could walk independently (GMFCS I+II) and 231 children (64%) who were independent concerning manual ability in age-relevant daily activities (MACS I+II).

Thirty-five children (10%) had both a severely limited self-mobility (GMFCS V) and did not handle objects (MACS V).

Of the 121 children with spastic hemiplegic CP 65 (54%) could walk in all environments and climb stairs (GMFCS I) as well as handle objects easily (MACS I). Two children in this group could only walk with an assistive device (GMFCS III) but none were evaluated as GMFCS IV-V or MACS V. There were 14 children with spastic hemiplegic CP (12%) who were not independent in their daily age-relevant manual activities (MACS III-IV).

In the group of children with spastic bilateral CP (n = 162) there was a large variation in gross motor function and manual ability. All 17 children with spastic tetraplegic CP were evaluated as GMFCS V and 15 also as MACS V. The children with diplegic CP (n = 145) were distributed between all levels of GMFCS and MACS.

In dyskinetic CP (n = 51), 34 children (67%) had a very limited self-mobility (GMFCS IV-V) as well as a limited manual ability (MACS IV-V).

The overall agreement between GMFCS and MACS was poor (kappa value 0.35, 95% confidence interval 0.27–0.41). Children with spastic hemiplegic CP had a significantly lower level of manual ability than gross motor function (Wilcoxon signed ranks test: p < 0.001, Sign test: p < 0.001). The reverse was found in children with diplegic CP (Wilcoxon signed ranks test: p < 0.001, Sign test: p < 0.001). In children with dyskinetic CP, no significant difference between GMFCS and MACS levels was found (Wilcoxon signed ranks test: p = 0.43, Sign test: p = 0.19).

## Discussion

This is, to our knowledge, the first study of the association between gross motor function (GMFCS) and manual ability (MACS) in a total population of children with CP. We found a poor overall correlation between the two systems as evaluated by the kappa statistics. However, in the CP subtypes, different associations were found. In hemiplegic CP, manual ability was more limited than gross motor function. The opposite was found in children with diplegic CP where gross motor function was more limited. We found a closer association between levels in children with dyskinetic CP.

In a previous study by Beckung and Hagberg [2], a strong correlation was found between GMFCS level and Bimanual Fine Motor Function (BFMF) level in a population of 178 children with CP. The BFMF has not been tested for reliability and validity and has more focus on manipulation and grip than on manual ability in daily activities. It is therefore difficult to compare BFMF levels with MACS levels, as the two systems are almost certainly describing very different aspects of function, with BFMF looking more at "impairment" and MACS more at "activity".

In the present study, the proportion of children with minor functional limitations, GMFCS I and MACS I, was higher than in some earlier studies [11,13,22]. We believe that this is mainly due to our careful inventories in order to identify all children with CP living in our region, to offer them participation in the CPUP programme. The prevalence of CP in the study area is 2.4/1.000 [23].

Gross motor function and manual ability in cerebral palsy are not equivalent entities. Hand function is very closely dependent on cognitive ability and voluntary motor control, and there is often a significant difference between maximal capacity and spontaneous performance, i.e., what the child *can do *and what he or she really *does*. The MACS aims to evaluate the latter and give a picture of how well the child can manage in daily manual activities. Performance and capacity are often more closely related in gross motor function, i.e., if a child *can *walk or sit, he or she usually *does *[24].

Another difference between gross motor function and upper limb function is the consequences of a unilateral impairment. A person with unilateral CP will probably walk, or try to walk, on the plegic leg but will sometimes not use the affected arm at all, leading to a lack of bimanual function. To be classified as MACS level I, bimanual hand function is required, and since many children with spastic hemiplegia use alternative strategies to compensate for poor bimanual function they will be evaluated as MACS level II.

These differences may explain why many children with hemiplegia in the present study were evaluated as functionally more limited in manual ability than in gross motor function. In children with spastic hemiplegic CP 53 of 121 (44%) were more limited in manual ability than in gross motor function.

Among children with diplegia 39% were more limited in gross motor function (GMFCS) than manual ability (MACS). The variability was large in this group, with a distribution across all levels of both the MACS and the GMFCS. This variability indicates that the CP subtype spastic diplegia alone gives us insufficient information about the child's gross motor function and about manual ability [25]. Almost all children with spastic tetraplegia were classified as GMFCS V and MACS V, and this subtype seems to be well defined in the Swedish classification. The term bilateral CP, suggested by the SCPE group, joins together spastic diplegia and tetraplegia, which further stresses the need for additional functional grading in order to correctly evaluate each child. A structured model for such a functional grading was also suggested by the SCPE group and may be very useful when describing populations of children with CP.

Children with dyskinetic CP in the present study (n = 51) had large functional limitations and 67% were at levels GMFCS and MACS IV and V. In this group of children, the association between GMFCS and MACS levels was strong. Children with ataxia constituted a small group in the present study (7%). Most had good motor function, 9 of 17 were GMFCS and MACS level I.

There were few outliers. Two children with MACS IV and GMFCS I had spastic hemiplegia, probably with decreased cognitive ability. Two children with MACS I and GMFCS IV had spastic diplegia, and one boy with MACS I and GMFCS V had dyskinetic CP.

The two different non-parametric tests yielded consistent results regarding the association between GMFCS and MACS in the CP subtypes. The Sign test is adequate for paired ordinal data, but generally has low statistical power. The Wilcoxon signed ranks test uses more information, but its applicability for ordinal data could be questioned.

## Conclusion

In the present study, we were able to demonstrate different patterns of gross motor function and manual ability in the different CP subtypes. Our results stress the importance of joining together information about the CP subtype, based on predominant neurological findings and functional evaluations. The GMFCS and the MACS seem to work well in this context and are both very useful in describing motor function characteristics in populations of children with CP.

## Abbreviations

CP = Cerebral Palsy

CPUP = A health care programme for children with cerebral palsy

GMFCS = Gross Motor Function Classification System

MACS = Manual Ability Classification System

SC = Swedish Classification of subtypes of Cerebral Palsy (see text)

SCPE = Surveillance of Cerebral Palsy in Europe network (see text)

## Competing interests

The author(s) declare that they have no competing interests.

## Authors' contributions

The three authors planned, performed and analysed the results of the study. KDC wrote the first draft of the manuscript, which was then actively improved and revised by all three authors. All authors read and approved the final manuscript.

**Table 3:** Distribution of children (n = 359) between levels of gross motor function (GMFCS) and manual ability (MACS) in relation to CP subtypes based on the Swedish classification system (SC) and the Surveillance of Cerebral Palsy in Europe (SCPE).

**SCPE**	**SC **	**MACS: I**	**II**	**III**	**IV**	**V**
**GMFCS I**						
**Spastic**	**Spastic**					
unilateral	hemiplegic	61	39	5	2	-
bilateral	tetraplegic	-	-	-	-	-
	diplegic	35	8	3	-	-
**Dyskinetic**	**Dyskinetic**					
Dystonic	Dystonic (tonus changing)	-	-	-	-	-
Choreo-athetotic	Athetotic	4	-	-	-	-
**Ataxic**	**Ataxic**	11	1	1	-	-
**Unclassified**	**Unclassified/Mixed**	1	1	-	-	-

**GMFCS II**						
**Spastic**	**Spastic**					
unilateral	hemiplegic	4	3	5	-	-
bilateral	tetraplegic	-	-	-	-	-
	diplegic	14	7	5	-	-
**Dyskinetic**	**Dyskinetic**					
Dystonic	Dystonic (tonus changing)	-	1	-	-	-
Choreo-athetotic	Athetotic	-	1	-	-	-
**Ataxic**	**Ataxic**	4	2	1	-	1
**Unclassified**	**Unclassified/Mixed**	-	-	-	-	-

**GMFCS III**						
**Spastic**	**Spastic**					
unilateral	hemiplegic	-	-	-	2	-
bilateral	tetraplegic	-	-	-	-	-
	diplegic	13	10	6	3	-
**Dyskinetic**	**Dyskinetic**					
Dystonic	Dystonic (tonus changing)	-	-	3	3	-
Choreo-athetotic	Athetotic	-	1	-	-	-
**Ataxic**	**Ataxic**	1	1	2	-	1
**Unclassified**	**Unclassified/Mixed**	-	-	-	-	-

**GMFCS IV**						
**Spastic**	**Spastic**					
unilateral	hemiplegic	-	-	-	-	-
bilateral	tetraplegic	-	-	-	-	-
	diplegic	2	4	8	8	6
**Dyskinetic**	**Dyskinetic**					
Dystonic	Dystonic (tonus changing)	-	1	1	2	8
Choreo-athetotic	Athetotic	-	-	-	3	2
**Ataxic**	**Ataxic**	-	-	-	2	-
**Unclassified**	**Unclassified/Mixed**	-	-	1	2	1

**GMFCS V**						
**Spastic**	**Spastic**					
unilateral	hemiplegic	-	-	-	-	-
bilateral	tetraplegic	-	-	1	1	15
	diplegic	-	-	-	-	2
**Dyskinetic**	**Dyskinetic**					
Dystonic	Dystonic (tonus changing)	1	-	1	3	15
Choreo-athetotic	Athetotic	-	-	-	-	1
**Ataxic**	**Ataxic**	-	-	-	-	-
**Unclassified**	**Unclassified/Mixed**	-	-	-	-	2

**Figure 3:**
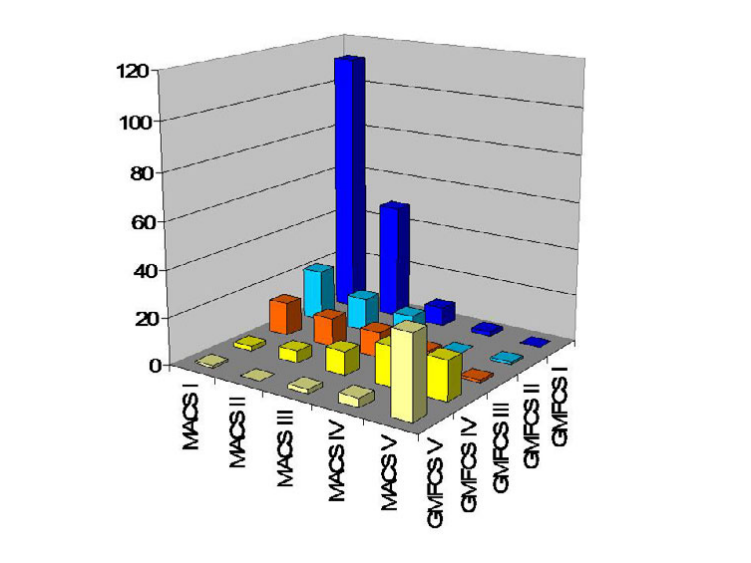
Association between GMFCS and MACS in the total population of children with CP.

## Pre-publication history

The pre-publication history for this paper can be accessed here:


